# Supplementary material for: End Sequence Analysis Toolkit (ESAT) expands the extractable information from single-cell RNA-seq data
Source: Genome Res. 2016 Oct;26(10):1397–410. doi: 10.1101/gr.207902.116 (PMC5052061; doi:10.1101/gr.207902.116)
Supplement: Supplemental Material [file supp_gr.207902.116_Supp_Materials.tar.gz › ESAT-esat_v0.1/doc/html/classbroad_1_1pda_1_1geneexpression_1_1clustering_1_1_hierarchical_clustering.html]

ESAT: broad.pda.geneexpression.clustering.HierarchicalClustering Class Reference


ESAT


- Main Page
- Related Pages
- Packages
- Classes
- Files

- Class List
- Class Index
- Class Hierarchy
- Class Members

All Classes Namespaces Files Functions Variables Enumerator Pages

- broad
- pda
- geneexpression
- clustering
- HierarchicalClustering

Public Member Functions |
Static Public Member Functions |
List of all members

broad.pda.geneexpression.clustering.HierarchicalClustering Class Reference

Collaboration diagram for broad.pda.geneexpression.clustering.HierarchicalClustering:

[legend]

|  |  |
| --- | --- |
| Public Member Functions | |
|  | HierarchicalClustering (MatrixWithHeaders data) |
|  | |
|  | HierarchicalClustering (MatrixWithHeaders data1, Map< String, Collection< String >> columnGroups, Map< String, Collection< String >> rowGroups) |
|  | |
| void | write (String save) throws IOException |
|  | |
| void | setClusterDistanceFunction (ClusterDistanceFunction distanceFunction) throws IllegalArgumentException |
|  | |
| void | setClusterDistanceFunction (String distanceFunctionName) |
|  | |
| void | setLinkage (String linkage) throws IllegalArgumentException |
|  | |
| void | cluster (boolean clusterRow, boolean clusterColumn) |
|  | |
| MatrixWithHeaders | getMatrix () |
|  | |

|  |  |
| --- | --- |
| Static Public Member Functions | |
| static void | main (String[] args) throws IOException, ParseException |
|  | |

## Constructor & Destructor Documentation

|  |  |  |  |  |  |
| --- | --- | --- | --- | --- | --- |
| broad.pda.geneexpression.clustering.HierarchicalClustering.HierarchicalClustering | ( | MatrixWithHeaders | *data* | ) |  |

Here is the caller graph for this function:

|  |  |  |  |
| --- | --- | --- | --- |
| broad.pda.geneexpression.clustering.HierarchicalClustering.HierarchicalClustering | ( | MatrixWithHeaders | *data1*, |
|  |  | Map< String, Collection< String >> | *columnGroups*, |
|  |  | Map< String, Collection< String >> | *rowGroups* |
|  | ) |  |  |

## Member Function Documentation

|  |  |  |  |
| --- | --- | --- | --- |
| void broad.pda.geneexpression.clustering.HierarchicalClustering.cluster | ( | boolean | *clusterRow*, |
|  |  | boolean | *clusterColumn* |
|  | ) |  |  |

Here is the caller graph for this function:

|  |  |  |  |  |
| --- | --- | --- | --- | --- |
| MatrixWithHeaders broad.pda.geneexpression.clustering.HierarchicalClustering.getMatrix | ( |  | ) |  |

|  |  |  |  |  |  |
| --- | --- | --- | --- | --- | --- |
| static void broad.pda.geneexpression.clustering.HierarchicalClustering.main | ( | String[] | *args* | ) | throws IOException, ParseException |

static

Here is the call graph for this function:

|  |  |  |  |  |  |
| --- | --- | --- | --- | --- | --- |
| void broad.pda.geneexpression.clustering.HierarchicalClustering.setClusterDistanceFunction | ( | ClusterDistanceFunction | *distanceFunction* | ) | throws IllegalArgumentException |

Here is the caller graph for this function:

|  |  |  |  |  |  |
| --- | --- | --- | --- | --- | --- |
| void broad.pda.geneexpression.clustering.HierarchicalClustering.setClusterDistanceFunction | ( | String | *distanceFunctionName* | ) |  |

Here is the call graph for this function:

|  |  |  |  |  |  |
| --- | --- | --- | --- | --- | --- |
| void broad.pda.geneexpression.clustering.HierarchicalClustering.setLinkage | ( | String | *linkage* | ) | throws IllegalArgumentException |

|  |  |  |  |  |  |
| --- | --- | --- | --- | --- | --- |
| void broad.pda.geneexpression.clustering.HierarchicalClustering.write | ( | String | *save* | ) | throws IOException |

---

The documentation for this class was generated from the following file:

- java/broad/pda/geneexpression/clustering/HierarchicalClustering.java


---

Generated on Fri Aug 15 2014 15:11:01 for ESAT by  

 1.8.7
